# Current Status and Future Perspective in Glioma Invasion Research

**DOI:** 10.3390/brainsci14040309

**Published:** 2024-03-26

**Authors:** Takanori Ohnishi

**Affiliations:** Department of Neurosurgery, Washoukai Sadamoto Hospital, Advanced Brain Disease Center, 1-6-1 Takehara, Matsuyama 790-0052, Japan; takanoriohnishi0024@gmail.com or tohnishi@m.ehime-u.ac.jp

## 1. Introduction

Glioblastoma (GBM) is the most malignant brain tumor in adults and shows an extremely poor prognosis, with a median survival of 15 months [1]. The main reason for this poor survival is the highly invasive nature of the tumor. Overcoming this invasiveness would greatly benefit patients with GBM, conferring much longer survival. GBM invasion causes poor prognosis via glioma cells that have infiltrated into peritumoral normal brain tissue escaping tumor resection and subsequent chemoradiotherapy, leaving tumor cells to allow for early tumor recurrence. In particular, glioma stem-like cells (GSCs) as glioma-initiating cells residing in the tumor border niche can diffusely migrate to normal brain tissue around the tumor mass and acquire much higher resistance to radio–chemotherapy by promoting a phenotypic transition to a mesenchymal type [2].

The molecular mechanisms underlying GBM invasion need to be better understood to establish novel treatment methods against GBM invasion. Many studies have investigated methods to control GBM invasion, but no reports have described the successful translation of such studies to effective therapy in clinical situations. Research in expectation of the development of clinically effective anti-invasion therapies uses two main approaches. The first approach involves basic studies to elucidate molecular mechanisms in the cellular processes of GBM invasion. To date, many studies related to molecular and biological mechanisms underlying glioma invasion have been performed, and the evidence accumulating from such studies contributes to a better understanding of the mechanisms underlying GBM invasion. Recently, the significance of the tumor microenvironment (TME) has been recognized as a factor promoting glioma invasion [3,4]. The TME comprises various elements, including nontumor cells such as neurons, astrocytes, and immune cells, along with the extracellular matrix, various growth factors, cytokines, and so on. Cellular behaviors such as the invasion, migration, and proliferation of tumor cells are performed in a harmonized fashion by crosstalk between tumor cells and elements of the TME to achieve the specific cellular events mentioned above.

The second approach involves clinical studies detecting how tumor cells infiltrating peritumoral normal brain tissue can be detected and removed. Various analytical methods using imaging modalities to preoperatively determine the extent of glioma invasion around the contrast-enhancing tumor mass of GBM have been reported. Such imaging modalities include ^11^C-methionine positron emission tomography (Met-PET), magnetic resonance (MR) spectroscopy, and chemical exchange saturation transfer (CEST) imaging [5,6]. In addition, the maximal resection of infiltrating tumors using special surgical procedures is important. The method most frequently used in GBM resection is 5-aminolevulinic acid (ALA)-guided surgery, which has been shown to significantly extend the overall survival of GBM patients compared to surgical resection without 5-ALA [7,8].

This Special Issue, titled “Special Issue: Advance in Glioma Invasion”, includes three review articles concerning the basic invasive mechanisms present in GBM, focusing on the TME and current and future anti-invasion therapies, in which special molecules in the TME become the targets of therapy against the GBM. The remaining three articles deal with an in vivo invasion model using zebrafish larvae, MR imaging for detecting nonenhancing areas in GBM, and a clinical study of the antitumor effects of carmustine (BCNU) wafers on GBM invasion. In this Editorial, I provide a brief overview of these articles in the next section and discuss the current status of glioma invasion and future directions of research that should be translated to clinically effective therapy against GBM invasion.

## 2. An Overview of Published Articles

Onishi et al. (Contribution 1) reviews the molecular mechanisms of glioma invasion based on cell adhesion to the extracellular matrix (ECM). The ECM is a major component of the TME and plays roles in scaffolding and the maintenance of tissue homeostasis. Degradation and remodeling of the ECM are essential events in the invasion and progression of cancer cells, including GBM. The adhesion and migration of tumor cells are promoted in a cooperative manner by various molecules in the ECM, including proteoglycans, fibronectin, laminin, cadherin, hyaluronic acid, and matrix metalloproteinases (MMPs). These molecules bind to their specific receptors and molecules in the ECM, promoting the migration and invasion of tumor cells. A TME showing hypoxic conditions and growth factors influences the cellular processes. In contrast, current antiglioma therapy, including radiation therapy, chemotherapy with temozolomide, and antivascular endothelial growth factor (VEGF) therapy, may change the TME to increase tumor invasiveness. Based on recent advances in the understanding of invasion mechanisms in glioma, molecularly targeted therapies have now been developed, but effective methods remain unclear.

Tamai et al. (Contribution 2) also mentions the significance of the TME in glioma invasion as a factor deeply related to the cellular processes in glioma invasion. Glioma invasion is enhanced by various factors that are presented in the TME. These include molecules and nontumor cells such as hypoxia-inducible factors (HIFs), ECM components, proteolytic enzymes (e.g., MMPs, membrane-type [MT]-MMP, tissue inhibitor of MMP [TIMP]), astrocytes, microglia, and macrophages. In addition, functions of the TME can be altered by various therapies, including radiation and chemotherapy, resulting in an increase in glioma invasiveness. Severe hypoxia upregulates HIF-1α expression, and this elevated HIF-1α activates the HIF-1α-related gene, increasing glioma invasion. ECM components such as hyaluronic acid (HA), fibronectin, tenacin-C, and proteolytic enzymes such as MMPs, MT-MMP, and a disintegrin and metalloproteinase promote glioma invasion, whereas TIMP and testican-3 inhibit invasion. Astrocytes in the TME are transformed to reactive astrocytes by direct contact with GBM cells, increasing the malignancy of GBM by facilitating tumor migration, invasion, and proliferation. Reactive astrocytes are induced by the activation of nuclear factor (NF)-κB signaling and sonic hedgehog-Gli signaling, upregulating signaling pathways such as the Janus kinase and signal transducer and activator of transcription (STAT) signaling and NF-κB pathway, resulting in the promotion of glioma cell invasion. Microglia and macrophages become glioma-associated microglia/macrophages by contact with tumor cells and play major roles in glioma invasion, angiogenesis, and immunosuppression. From this perspective, the authors suggest that anti-invasion therapy provided based on these mechanisms of glioma invasion may indicate that normal brains tend to scatter tumor cells within normal brain tissues. This may mean that the best therapy for GBM patients may be to promote the coexistence of infiltrating glioma cells with peritumoral normal brain areas.

Nakahara et al. (Contribution 3) reviewed the role of the tumor suppressor gene N-myc downstream-regulated gene 1 (*NDRG1*) in tumorigenesis and the acquisition of resistance to anti-GBM therapies for gliomas and glioblastoma. This review analyzes the expressions and functions of *NDRG1* in GBM by searching the PubMed and Scopus databases. NDRG1 is expressed in both normal and cancer cells. In cancer cells, NDRG1 has inhibitory effects on cell proliferation, invasion, migration, metastasis, and angiogenesis and promotes apoptosis and differentiation. *NDRG1* is affected by hypoxia to significantly increase the expression of the NDRG1 protein in GBM cells in the hypoxic state. Although the exact cellular functions of NDRG1 protein have not been elucidated, mutations in *NDRG1* or the aberrant expression of the NDRG1 protein were demonstrated to be associated with tumor-suppressive and oncogenic phenotypes.

To elucidate the mechanisms underlying the cellular processes of glioma invasion, in vitro and in vivo models of tumor invasion are essential. As invasion of glioma cells occurs in the normal brain, and in vivo tumor xenograft mouse models can present invasive processes under conditions close to those in human brains, but these studies are often complex, time-consuming, and associated with difficulty in exploring key molecules that play a crucial role in GBM invasiveness [9]. Consequently, new in vitro tumor models that can incorporate multiple cell types, ECM materials, and spatial and temporal administration of soluble factors, particularly the incorporation of factors stimulating the tumor vasculature, have been developed [10]. However, even in these well-designed in vitro three-dimension (3D) models, interactions of tumor invasion and the TME cannot be visualized in the same way as a living condition. 

Larsson et al. (Contribution 4) present an in vivo xenograft zebrafish model by orthotopically injecting cultured stem cells of pediatric high-grade glioma into the brains of zebrafish larvae. This in vivo model offers advantages to monitor the cellular processes underlying tumor invasion for a relatively long time and to quickly evaluate the effects of drug treatment. This in vivo 3D analysis model allows for not only a visualization of the number, volume, and morphology of tumor cells injected into fish brains using in vivo confocal microscopy but also an analysis of the alterations in morphology, differences in growth patterns, and degrees of migration and invasion in different tumor cell lines. In addition, the results of studies using this orthotopic zebrafish model are confirmed to show the same results of in vitro invasion studies of GSCs in cell culture and in vivo studies of GSC invasion using xenotransplantation into mice brains, indicating that the present model represents actual cell behaviors. One limitation of this model lies in the procedure, since the injection of cells into the small brains of zebrafish requires high-level technical skills.

Yamamoto et al. (Contribution 5) report a new method in which a nonenhancing tumor in GBM is predicted and visualized via the T1w/T2w ratio map in MRI. The ratio of T1- to T2-weighted images (rT1/T2) is reported as an imaging surrogate for the microstructures of the brain. In this study, using rT1/T2 as a surrogate for the T1 and T2 relaxation time of GBM, the possibility of visualizing a nonenhancing tumor in GBM is investigated. Thirty-four GBM patients are analyzed in terms of relationships between rT1/T2 values and areas of positive methionine (Met) uptake with a tumor-to-normal-tissue ratio (T/N ratio) >1.5 on ^11^C-Met positron emission tomography (Met-PET) and areas of T2-FLAIR hyperintense lesion. Values of rT1/T2 correlate significantly with both T1 and T2 relaxation times in a logarithmic manner. Values of rT1/T2 show significance for detecting Met-PET high and T2-FLAIR hyperintense lesions. These results may imply that rT1/T2 holds promise as an imaging marker for detecting nonenhancing tumors in GBM.

Ohnishi et al. (Contribution 6) report a clinical study investigating the antitumor effects of interstitial chemotherapy with BCNU wafers, particularly antitumor effects on invasive tumor cells in normal tissue around the resection cavity wall in GBM. To investigate how BCNU released from the wafer has antitumor effects on GBM, concentrations of BCNU in cerebrospinal fluid are measured through the Ommaya reservoir placed in the resection cavity from immediately after implantation of the wafer on the brain surface of the resection cavity to 30 days postoperatively. Areas under the curve (AUC) for BCNU are calculated in each patient. GBM patients are divided into two groups according to the cut-off value for median progression-free survival: early recurrence (ER) and late or no recurrence (LN). In addition, all patients are classified into two phenotypes according to the MRI and Met-PET findings: highly invasive and less invasive. All ER patients show highly invasive types of GBM. The AUC for BCNU does not differ significantly between the ER and LN groups. Total BCNU concentrations do not correlate with tumor progression or survival, but sustained high concentrations of extracellular BCNU may have some potential to provide a survival benefit for patients with less-invasive-type GBM.

## 3. Discussion

The mechanisms of GBM invasion have yet to be completely clarified, but much evidence has been accumulated to date. Recent studies have demonstrated that interactions of tumor cells and the TME play a critical role in the processes of tumor invasion. These interactions regulate tumor invasive intensity, speed, and direction of tumor cell migration, and the activation of molecules according to these processes.

**Figure 1:**
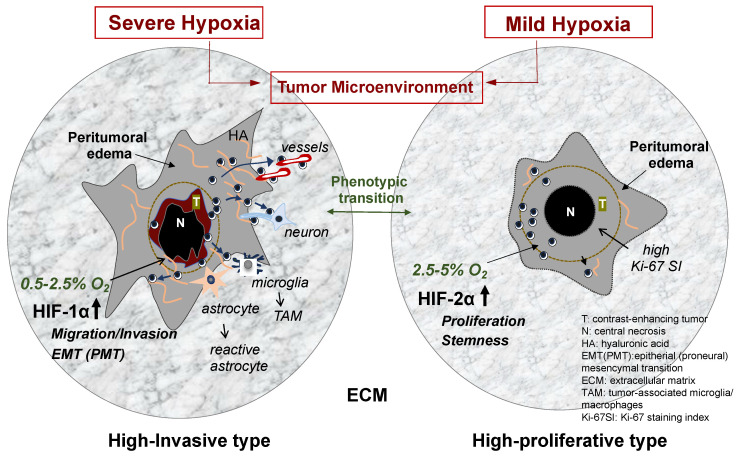
GBM phenotypes based on differential hypoxia in the tumor microenvironment. When oxygen concentration in the tumor periphery is very low (severe hypoxia of 0.5–2.5% O_2_), HIF-1α is upregulated, and then the transcription of HIF-1α-related genes is activated. These include many genes that promote cell migration, invasion, and phenotypic transition to mesenchymal phenotypes. The tumor shows highly invasive phenotype in the biological features and imaging findings on MRI (left illustration). In contrast, when the concentration in the tumor periphery is mildly low (moderate–mild hypoxia of 2.5–5% O_2_), HIF-2α is upregulated, and then the transcription of HIF-2α-related genes is activated. These include genes mainly participating in tumor cell proliferation and maintenance of stemness of glioma stem cells. The tumor presents less invasive and highly proliferative features and a well demarcated tumor imaging on MRI (right illustration). Glioma cell invasion is induced by various elements in the TME under hypoxia. Among them, ECM plays a crucial role in the cellular processes of glioma invasion. The illustration displays various elements in the TME, which promote glioma invasion. These include ECM components and preferential guidance for glioma invasion and nontumor cells such as astrocytes and microglia.

Tumor cell migration and invasion are highly integrated events with multistep processes that are executed in a coordinated manner through the interactions of tumor cells and components of the TME. The variety of components of the TME participate in promoting and regulating glioma invasion (Figure 1).

Tumor cells primarily adhere to ECM components such as HA and proteoglycans which constitute scaffolds for migrating tumor cells in the extracellular space. They also adhere adjacent cells such as neurons, astrocytes, and endothelial cells of vessels which constitute so called “secondary structures” [11] promoting tumor invasion as preferential routes. The process requires various molecules in the TME to bind to specific receptors or adhesion molecules on tumor cells. For example, the binding of laminin and fibronectin to integrin receptors, HA to CD44 and Rhamm, and the homotypic binding of neuronal cell adhesion molecules (NCAM) and L1 on the surface of axonal fibers of neurons to the corresponding molecules on glioma cells.

How these interactions between tumor cells and the TME components are regulated in the processes of GBM invasion remains unclear. One example is that details remain unclear on how tumor cells use different signal pathways in a coordinated manner through the binding of HA and CD44 and that of fibronectin and laminin to integrins. Binder et al. reported that the migration and invasion of GBM cells are unaffected by the knockdown of integrin but are reduced by CD44 knockdown [12]. In addition, Kim et al. reported that the binding of CD44-HA-activated cell adhesion is achieved more rapidly but more weakly than that of integrin ligand-activated cell adhesion [13]. These results indicate that the cellular processes with CD44 and HA binding may have rapid, weak natures in cell adhesion, whereas cellular processes through the binding of integrins and their ligands may result in fewer, slower adhesion interactions but the reinforcement of CD44-HA interactions. HA is a major molecule that is widespread in the extracellular space of the brain, whereas collagen, fibronectin, and laminin are expressed on limited tissues such as the basal membrane of blood vessels and the pial membrane. These results indicate the specific binding of cell receptors to their ligands in the ECM, which may provide critical information to achieve specific cellular behaviors.

These interactions of tumor cells and the TCM are influenced by various factors, including oxygen concentration, acidity, and microglia/macrophages. Among these, hypoxia in the TCM is well known to modify various cellular processes, including GBM invasion [3]. Some GBM cells, particularly glioma stem-like cells, reside in the tumor border niche under oxygen conditions of moderate-to-severe hypoxia. The degree of hypoxia differs between tumor regions. The tumor core shows very low oxygen tension (0.1–0.5% O_2_), whereas the tumor periphery presents severe hypoxia (0.5–2.5% O_2_) [14]. These differences in hypoxia induce the expression of hypoxia-inducible factors (HIFs). Among the HIFs, HIF-1a is upregulated by severe hypoxia (~1% O_2_), and HIF-2a is upregulated by moderate-to-mild hypoxia (2.5–5% O_2_) [15]. These differences in the degree of hypoxia in and around GBM may influence cellular processes in not only GBM invasion but also tumor progression and recurrence.

The entire picture of the mechanisms involved in GBM invasion remains undisclosed, but the interactions of various cells and molecules are understood to be complicated but coordinated to regulate the specific cellular behaviors under specific microenvironments, including hypoxia.

Another topic seeing advances in the understanding of glioma invasion is how glioma cells infiltrating normal brains around the tumor mass can be effectively detected and surgically resected. Previous studies have reported that maximized tumor resection, including infiltrating tumor cells, can improve prognosis for GBM patients [16]. As GBM is an invasive tumor, complete removal of a tumor that includes tumor cells that have infiltrated the normal brain has been considered impossible or difficult, particularly in eloquent areas. Consequently, the development of methods for preoperatively detecting infiltrating tumor cells and elaborate intraoperative techniques for finding and removing infiltrating tumor cells are required. The former includes detecting the extent of tumor cell invasion in peritumoral areas using various imaging modalities. The latter involves the detection of infiltrating tumor cells (in the form of GSCs) by fluorescence-guided surgery using markers such as 5-ALA linked to a neuronavigational system [17].

Recent studies have reported the advantage of Met-PET in detecting nonenhancing tumors infiltrating the peritumoral normal brain [18]. We have also reported the utility of Met-PET in identifying infiltrating tumor cells, including GSCs, in which the area with tumor infiltration was defined as the Met-uptake area with a T/N ratio ≥1.4 [19].

To make use of imaging studies for predicting areas of GBM invasion during tumor resection, novel methods superior to the present 5-ALA-guided surgery are required. Recently, in addition to the introduction of the spectroscopy of 5-ALA fluorescence intensity to detect tumor cells in peritumoral nonenhancing tumors, we identified a novel enhancer for 5-ALA-derived fluorescence intensity [20]. The molecule can enhance 5-ALA intensity not only in differentiated glioma cells but also in human GBM stem-like cells through the activation of 5-ALA synthetase 1. The introduction of this molecule in 5-ALA-guided surgery could provide a more distinguishable visualization of GSCs, resulting in more complete surgery and thus a reduced risk of remnant infiltrating GSCs.

## 4. Future Perspectives

High invasiveness of GBM is a characteristic feature that represents a major cause of poor prognosis in patients with GBM. Although the molecular mechanisms underlying GBM invasion have not been fully clarified, the interactions of various factors in the TME with tumor cells through cross-stalk have been demonstrated to be important. Among such factors, differential hypoxia plays a key role in the motile and invasive behavior of tumor cells, particularly GSCs in and around the tumor mass. As hypoxia can induce phenotypic transition from the proneural (epithelial) type to the mesenchymal type and vice versa, highly invasive types of GSCs could alter the phenotype to a highly proliferative type with decreasing invasiveness based on the “go-or-grow theory” [21]. The degree of hypoxia may thus regulate the invasive activity of GSCs, resulting in the promotion of tumor growth, as observed in tumor progression and recurrence. These cellular processes may indicate that the simple inhibition of glioma invasion might be inducible with aggressive tumor growth. If glioma cells can be kept moving in normal brain tissue, GBM patients may survive much longer as described in the perspectives of Tamai et al. (Contribution 2). In future, this matter needs to be investigated with continued research into the establishment of effective anti-invasion therapies.

## Data Availability

Not applicable.
